# Toll-like receptor 4 deficiency or inhibition does not modulate survival and neurofunctional outcome in a murine model of cardiac arrest and resuscitation

**DOI:** 10.1371/journal.pone.0220404

**Published:** 2019-08-01

**Authors:** Stefan Bergt, Andrea Grub, Melanie Mueller, Rika Bajorat, Ivan Barilar, Brigitte Vollmar, Jan Patrick Roesner, Nana-Maria Wagner

**Affiliations:** 1 Clinic for Anesthesiology and Critical Care Medicine, Rostock University Medical Center, Rostock, Germany; 2 Clinic for Anesthesiology, Intensive Care Medicine and Pain Therapy, University Hospital Muenster, Muenster, Germany; 3 Molecular and Experimental Mycobacteriology, Priority Area Infections, Research Center Borstel, Borstel, Germany; 4 Institute for Experimental Surgery, Rostock University Medical Center, Rostock, Germany; Indiana University School of Medicine, UNITED STATES

## Abstract

**Background:**

Patients experiencing cardiac arrest (CA) and cardiopulmonary resuscitation (CPR) often die or suffer from severe neurological impairment. Post resuscitation syndrome is characterized by a systemic inflammatory response. Toll-like receptor 4 (TLR4) is a major mediator of inflammation and TLR4 has been implicated in the pathogenesis of post-resuscitation encephalopathy. The aim of this study was to evaluate whether TLR4 deficiency or inhibition can modulate survival and neurofunctional outcome after CA/CPR.

**Methods:**

Following intubation and central venous cannulation, CA was induced in wild type (C57Bl/6J, n = 38), TLR4 deficient (TLR4^-/-^, n = 37) and TLR4 antibody treated mice (5mg/kg MTS510, n = 15) by high potassium. After 10min, CPR was performed using a modified sewing machine until return of spontaneous circulation (ROSC). Cytokines and cerebral TNFalpha levels were measured 8h after CA/CPR. Survival, early neurological recovery, locomotion, spatial learning and memory were assessed over a period of 28 days.

**Results:**

Following CA/CPR, all mice exhibited ROSC and 31.5% of wild type mice survived until day 28. Compared to wild type mice, neither TLR4^-/-^ nor MTS510 treated wild type mice had statistically significant altered survival following CA/CPR (51.3 and 26.7%, P = 0.104 and P = 0.423 vs. WT, respectively). Antibody-treated but not TLR4^-/-^ mice had higher IL-1β and IL-6 levels and TLR4^-/-^ mice had higher IL-10 and cerebral TNFalpha levels. No differences existed between mice of all groups in early neurological recovery, locomotion, spatial learning ability or remembrance.

**Conclusion:**

Therapeutic strategies targeting TLR4 may not be suitable for the reduction of mortality or neurofunctional impairment after CA/CPR.

## Introduction

The incidence of all-rhythm out of hospital cardiac arrest (OHCA) assessed by emergency medical services varies among countries and regions but is estimated as 73 in the U.S. and 84 per 100,000 population in Europe. Patients suffering from CA received cardiopulmonary resuscitation (CPR) by emergency medical services in 40.6 and 47.3 per 100,000 population, respectively. 29.0% of patients in the US and 25.2% in Europe survived until hospital admission and 10.8% of patients in the US and 10.3% in Europe until hospital discharge [1,2]. Of those discharged, the majority of patients suffers from severe disability with low potential for rehabilitation [3]. In long term follow ups up to 24 months, in addition to motor functional disability, all patients report cognitive deficits such as severe intellectual impairment, dementia or amnesic syndrome [4] and 70% cannot return to an independent way of life [5].

Although cardiac arrest is the initiating event, the degree of disability and mortality in patients undergoing CPR is primarily determined by the extend of injury to the brain [6]. Neuronal oxygen stores are depleted within 20sec after the onset of cardiac arrest and CPR can only maintain 30% of prearrest cerebral blood flow [7]. The initial circulation following the return of spontaneous circulation (ROSC) is mostly insufficient resulting in persisting hypoxia further contributing to ischemia induced neuronal cell damage [8,9]. Once hemodynamics and respiration suffice to fully restore oxygen supply, reperfusion injury occurs further aggravating tissue damage and cell death. Cerebral tissue necrosis leads to spill over of otherwise intracellular proteins such as heat shock proteins, hyaluronic acid, fibronectin and high mobility group box 1 (HMGB1) into the extracellular compartment that can become detectable in the plasma of patients after cerebral ischemia in the context of stroke or CA/CPR [10–12]. These proteins can bind and activate toll like receptors (TLRs) such as TLR2 and TLR4. Both TLRs are implicated in the progression of cerebral injury induced by ischemia and reperfusion and can be found upregulated in patients following CA/CPR [13,14].

For decades the mortality and disability rates in patients after CA/CPR has remained high with hypothermia being the only causative treatment option [15]. We here propose genetic ablation or pharmacological inhibition of TLR4 augments survival and neurofunctional outcome in mice after CA/CPR. To test this hypothesis we used a previously established, highly standardized model of CA/CPR employing a modified sewing machine to perform CPR after 10min of high potassium-induced cardiac arrest [16,17]. During a 28 day follow up period, we assessed survival and dissected neurofunction by specifically addressing early neurological rehabilitation, motor function, spatial learning ability and memory after CA/CPR.

## Methods

### Verification of the *Tlr4*^*LPS-del*^ spontaneous mutation and corresponding functional consequences in TLR4^-/-^ mice

All animal procedures were approved by the governmental ethical board (Landesamt für Landwirtschaft, Lebensmittelsicherheit und Fischerei Mecklenburg Vorpommern, LALLF 7221.3–1.1-022/11) in accordance with institutional, national and European guidelines for the care and use of laboratory animals. In the present study, B6.B10ScN-Tlr4^lps-del^ mice purchased from Charles River, Germany, were used to assess the effects of TLR4 in CA/CPR. In these animals, we verified the presence of the *Tlr4*^*Lps-del*^ spontaneous mutation corresponding to a 74723 bp deletion completely removing the *Tlr4* coding sequence resulting in a shortened mRNA product of 140 instead of 390bp which does not allow for functional TLR4 protein expression (S1 Fig). TLR4^-/-^ mice exhibit defective responses to lipopolysaccharide (LPS) stimulation and a reduced expression of proinflammatory genes.[18] Functional verification of TLR4 deficiency in TLR4^-/-^ mice revealed a reduced increase in interleukin-6 (IL-6) and interleukin-10 levels (IL-10, both detected in plasma by sandwich immune assay, R&D systems, Minneapolis, USA) 8h after intraperitoneal administration of 20 mg/kg bodyweight LPS (S2 Fig).

### Murine model of cardiac arrest and cardiopulmonary resuscitation

**Fig 1:**
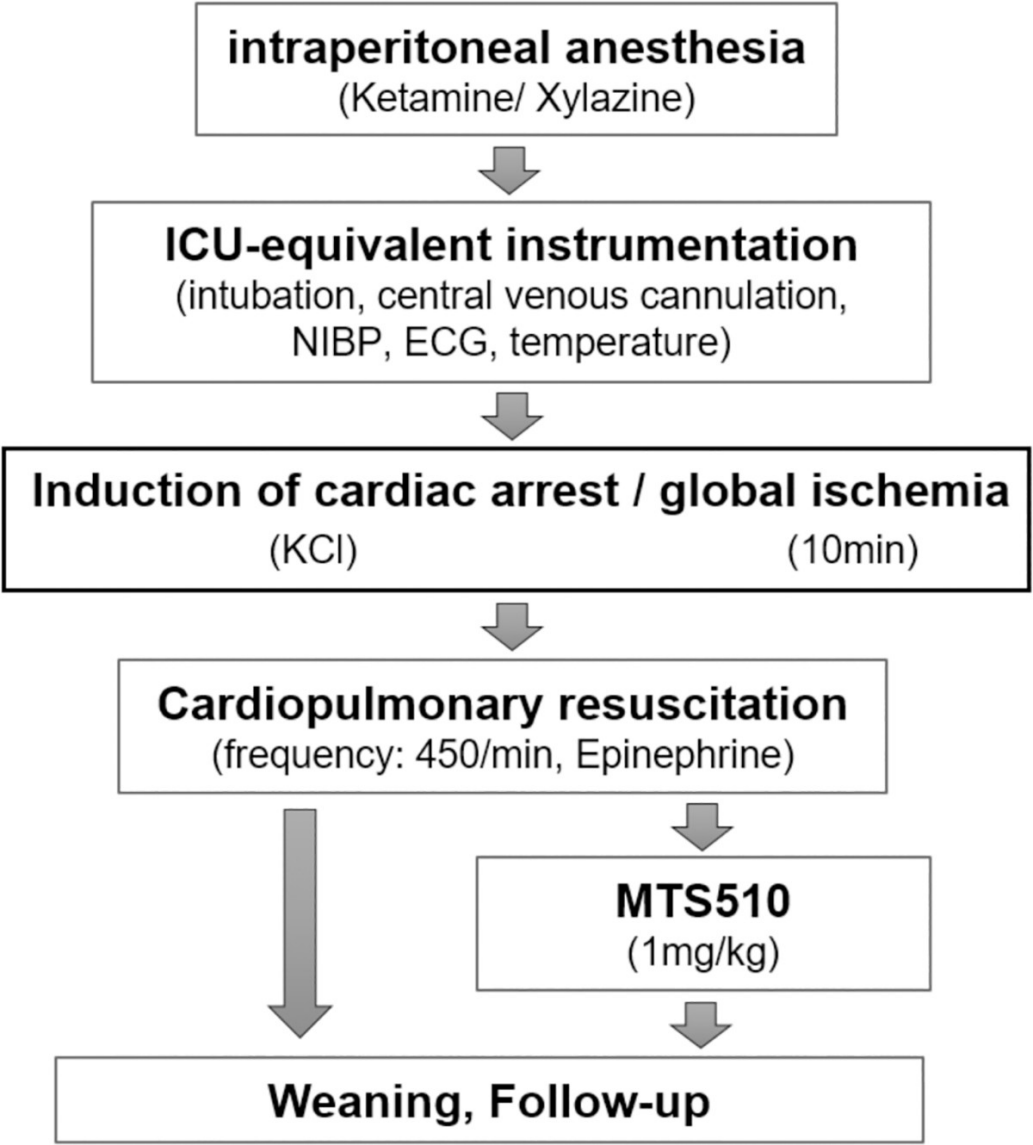
Protocol of cardiac arrest induction and cardiopulmonary resuscitation. Abbreviations: ICU intensive care unit, NIBP non-invasive blood pressure, ECG electro cardiogram, KCl potassium chloride.

The model of cardiac arrest (CA) and cardiopulmonary resuscitation (CPR) was conducted as described previously. [16,17] Only female mice were used since male mice almost exclusively presented with urinary outflow obstruction and death from post renal kidney failure between day 4 and 10 after CA/CPR.[16] 53 female C57BL/6J and 37 female B6.B10ScN-Tlr4^lps-del^ mice (12–16 weeks, 19–22 g) were anaesthetized by intraperitoneal injection of 12μg/g ketamine and 8μg/g xylazine and subjected to oral intubation and mechanical ventilation (0.21 inspired oxygen fraction). A central venous catheter was inserted into the right jugular vein, blood pressure was monitored non-invasively and ECG monitoring was initiated. CA was induced by injection of 80 μg/g potassium chloride and mechanical ventilation was interrupted upon verification of cardiac arrest by ECG. Resuscitation was initiated following 10min of CA, ventilation was resumed (220/min; FiO_2_ 1.0), precordial chest compressions were begun with a frequency of 450/min employing a modified sewing machine and 0.4 μg/g epinephrine were injected. At the beginning of CPR, a subset of n = 15 wild type mice received 1 mg/kg bodyweight MTS510, a rat monoclonal antibody reacting with murine TLR4 (abcam, Cat.-No. ab95562) via the jugular vein catheter.[19,20] Following 2min of CPR, FiO_2_ was reduced to 0.6 and returned to baseline (FiO_2_ 0.4) after 20min of successful resuscitation (Fig 1). One hour after return of spontaneous circulation (ROSC), the jugular vein catheter was removed, wounds were surgically ligated and mice weaned from mechanical ventilation. To prevent dehydration, mice received 0.5mL saline subcutaneously. All mice were weighed on the day of CPR (day 0, prior CA/CPR) as well as on each of the following 14 days and the day of the end of the observation period of the study (day 28). Following 10 min of cardiac arrest (CA), all mice exhibited return of spontaneous circulation (ROSC) following cardiopulmonary resuscitation (CPR) and epinephrine injection and were successfully weaned from mechanical ventilation. Heart rate, mean arterial blood pressure and body temperature were assessed 1 and 2h after CA/CPR and body temperature was monitored at 4, 8, 12 and 24h after CA/CPR in all animals.

### Assessment of cytokine release following CA/CPR

In a separate set of experiments using n = 8 mice per group, blood samples were drawn by puncture of the inferior caval vein, centrifugated and plasma was stored at -80°C pending analysis. Samples were analyzed for the presence of cytokines using commercially available kits for high mobility group box 1 (HMGB1, ELISA, ibl-international GmbH, Hamburg, Germany and Shino-Test corporation, Japan), interleukin-6 (IL-6), interleukin-1beta (IL1β) and interleukin-10 (all from Thermo Scientific, Pierce Biotechnology, Rockford, USA) and a microplate reader (Sunrise Remote, TECAN Austria GmbH, Salzburg, Austria). All data are presented as mean±SD.

### Analysis of TNFalpha levels after CA/CPR

In separate sets of experiments, n = 7 WT and n = 7 TLR4^-/-^ mice were sacrificed 8h after CA/CPR and n = 4 WT and n = 4 TLR4^-/-^ after sham-procedures and cerebral tissue was harvested and snap frozen at -80°C. Total RNA was extracted from brains, reverse-transcribed and analyzed for TNFalpha expression using the primer pair TCC CCA AAG GGA TGA GAA G (for) and GCA CCA CTA GTT GGT TGT C (rev). Expression levels of TNFalpha mRNA were normalized to Rps7-housekeeping gene, plotted as mean of 2^-ΔΔCt^ ± SEM and transformed relative to mean values of WT sham controls.

### Analysis of neurological function

Analysis of neurological function was performed as previously described [16,17]. NeuroScore: Level of consciousness, corneal reflex, respirations, righting reflex, coordination and movement/activity were assessed. Within each item, 0, 1 or 2 points were achievable resulting in 12 points representing the maximum score [21,22]. Assessment was performed by an unbiased observer. RotaRod test: Mice were subjected to balancing on a rotating cylinder (12,5 revolutions/min) for three attempts of 300sec (900sec in total) and the time until mice fell off the rod was recorded [23,24]. Both NeuroScore and RotaRod test were applied on the day of CA/CPR (d 0, 1 h prior to CA/CPR) as well as on each of the following days until day5 and on day7, 14 and 28 after CA/CPR. Water Maze test: Mice were trained daily (twice a day at 8am and 6pm, five attempts in each session) beginning on day5 until the day before CA/CPR to find a 5x5cm escape platform located 0.5cm below the water surface in a circular tank (60cm in diameter, 40cm in height) filled with water.[25] For the investigation of mice memory function following CA/CPR, the time required to find the platform was again measured when animals were placed at the same starting position within the tank. In order to avoid loss of animals from drowning due to general weakness of catabolic state, mice underwent the test after CA/CPR only when they a) had reached a maximum score in the NeuroScore, b) had fulfilled an attempt of 300 sec on the RotaRod and c) exhibited a halt in loss of body weight (day X following CA/CPR). The test was the performed daily until dayX+2. To investigate the capability of new spatial learning after CA/CPR, the position of the escape platform was randomly changed on day10 and the test was performed for the next 5 days in the same manner like before CA/CPR.

### Statistical analysis

Statistical analysis was performed employing Sigma Plot 10 (Jandel Corporation, San Rafael, CA, USA), R 3.6.0 (R Core Team) and Graph Pad Prism 6.0 (La Jolla, CA, USA) and statistical significance was defined as *P<0*.*05*. Survival R package was used to perform Kaplan-Meier Survival Analysis and the resulting survival curves were compared with a log-rank test. Based on the obtained results hazard ratios for the TLR4^-/-^ and MTS 510 groups were computed using the Cox proportional hazards regression model and a post hoc estimate of sample size needed to obtain significant differences in survival rates was made with the powerSurvEpi R package. Survival analysis results were visualized with the survminer R package. Time of cardiac arrest was defined as the start point for survival analysis, all animals could be resuscitated and were followed for 28 days or until death. Results from the Water Maze test were analyzed using Kolmogorov-Smirnov test for normal distribution and Wilcoxon matched-pairs signed-rank test. For analysis of quantitative data, student´s t-test was used for the comparison of two groups and ANOVA followed by Bonferroni correction for multiple comparisons for comparison of three or more groups. A numeric difference in participants on Water Maze test after CA/CPR was evaluated by Chi-Square test.

## Results

### Hemodynamic and physical parameters at baseline and after CA/CPR

Bodyweight, heart rate, mean arterial pressure and body temperature of mice were comparable between wild type, TLR4^-/-^ and MTS 510 treated wild type mice before CA/CPR or sham treatment (Table 1). Mice received comparable amounts of epinephrine and all mice exhibited return of spontaneous circulation (ROSC) after CA/CPR with no significant differences between groups in the time required until ROSC occurred. The time needed to successfully wean animals from mechanical ventilation were equally comparable between groups. 1 and 2 hours after CA/CPR, no significant differences among heart rate, mean arterial blood pressure or body temperature were observed between groups. Body temperature was comparable at 4, 8, 12 and 24h after CA/CPR in all groups. A minority of animals (n = 4 in the wild type group, n = 4 TLR4^-/-^ and n = 2 MTS 510 treated wild type animals) exhibited seizures and n = 5, n = 4 and n = 1 irreversible paresis of hindlimbs, respectively (Table 1).

**Table 1:** Baseline hemodynamic and physical parameters.

	WT	TLR4^-/-^	WT + MTS 510
	n = 38	n = 37	n = 15
**baseline before preparation for CA/CPR**			
body weight [g]	20,67 [± 2,0]	22,08 [± 3,1]	20,44 [± 1,4]
heart rate [1/min]	244 [± 32]	233 [± 23]	241 [± 31]
MAP [mm Hg]	101 [± 11]	90 [± 11]	94 [± 8]
body temperature [°C]	36,0 [± 0,1]	36,0 [± 0,1]	36,0 [± 0,1]
**parameter of CA/CPR**			
ROSC time [s]	84 [± 25]	77 [± 20]	65 [± 22]
ROSC rate	100%	100%	100%
Adrenalin (μg)	15,0 [10 – 15]	12,5 [10 – 15]	12,5 [10 – 15]
weaning (min)	140 [± 12]	137 [± 10]	136 [± 8]
**1 hour after CA/CPR**			
heart rate [1/min]	429 [± 37]	457 [± 26]	440 [± 42]
MAP [mm Hg]	67 [± 8]	63 [± 12]	71 [± 10]
body temperature [°C]	36,4 [± 0,2]	36,0 [± 0,3]	36,2 [± 0,1]
**2 hours after CA/CPR**			
heart rate [1/min]	277 [± 27]	301 [± 33]	287 [± 27]
MAP [mm Hg]	60 [± 10]	65 [± 15]	64 [± 12]
body temperature [°C]	36,0 [± 0,2]	36,0 [± 0,1]	36,1 [± 0,2]
**observation period after CA/CPR**			
body temperature			
4 h post CA/CPR [°C]	35,4 [± 0,8]	35,0 [± 0,9]	35,7 [± 0,5]
8 h post CA/CPR [°C]	35,2 [± 1,0]	34,8 [± 1,3]	35,1 [± 1,2]
12 h post CA/CPR [°C]	35,8 [± 0,9]	35,2 [± 0,8]	35,2 [± 0,9]
24 h post CA/CPR [°C]	35,0 [± 1,2]	35,4 [± 1,1]	35,1 [± 1,4]
adverse events			
seizures [n]	4	5	2
paresis of hind limbs			
irreversible [n]	5	4	1
reversible [n]	1	2	1

### Survival of WT, TLR4^-/-^ and TLR4-blocking antibody treated mice after CA/CPR

**Fig 2:**
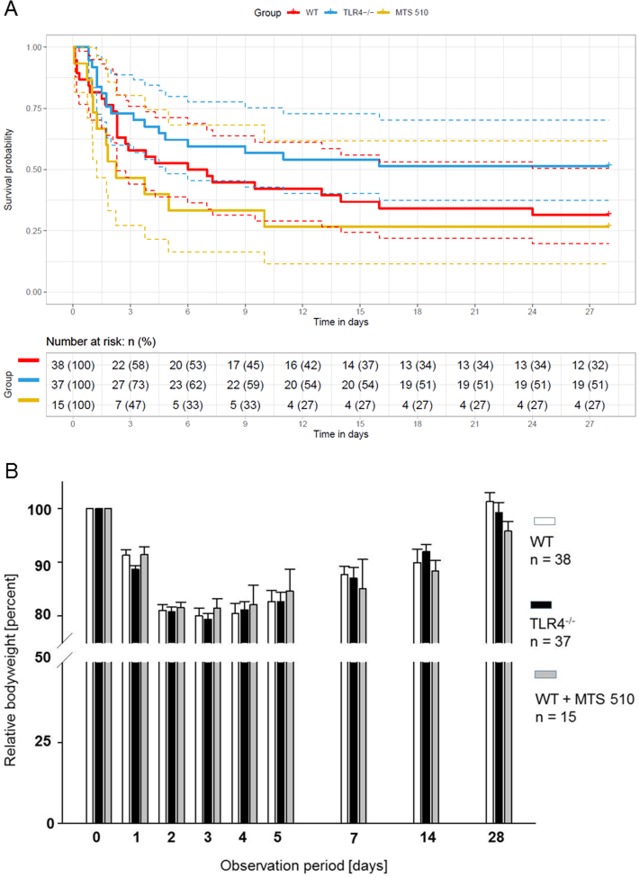
Survival and body weight over a 28 days observation period after CA/CPR. **(**A) Kaplan-Meier plot for mouse survival after CA/CPR. Red, blue and yellow full lines denote the WT (n = 38 mice), TLR4^-/-^ (n = 37) and MTS 510 (n = 15) survival curves while the dashed lines represent their 95% confidence intervals. The survival table depicts the number and percentage of animals alive at three-day intervals for each of the groups. No significant differences between the groups were detected (P = 0.104 for the comparison of WT vs. TLR4^-/-^ and P = 0.423 for the comparison of WT vs. MTS510-treated WT mice). (B) Body weight of the three treatment groups after CA/CPR. There were no significant differences between groups at any point of time.

Following CA/CPR, all mice exhibited ROSC. Heart rates, arterial blood pressure and body temperature were comparable between mice/groups (Table 1). During the 28 day observation period of the study, 31.5% of wild type mice survived. Of the TLR4^-/-^ mice, 51.3% survived and 26.7% of wild type mice treated with the TLR4 blocking antibody MTS 510 survived. Although more TLR4^-/-^ mice survived compared to wild type mice, this difference did not yield statistical significance (Fig 2A). Based on these results, hazard ratios were computed using the Cox proportional regression model (S3 Fig) and a post hoc estimate of sample size needed to obtain significant differences in survival was made. These analyses revealed that for comparisons with 80% power, 240 additional mice would be needed (122 WT and 118 TLR4^-/-^) to obtain P values of less than 0.05 for the comparison of survival of WT vs. TLR4^-/-^. For WT vs. antibody-treated WT mice, additional 643 mice (461 WT and 182 antibody-treated mice) would be required. Bodyweight is a sensitive parameter of overall wellbeing of the laboratory animal mouse [26]. In line with no significant differences in survival after CA/CPR, no differences in bodyweight were observed between wild type, TLR4^-/-^ and MTS 510 treated wild type mice (Fig 2B).

### Pro- and anti-inflammatory plasma cytokine levels and cerebral TNFalpha expression 8h after CA/CPR

**Fig 3:**
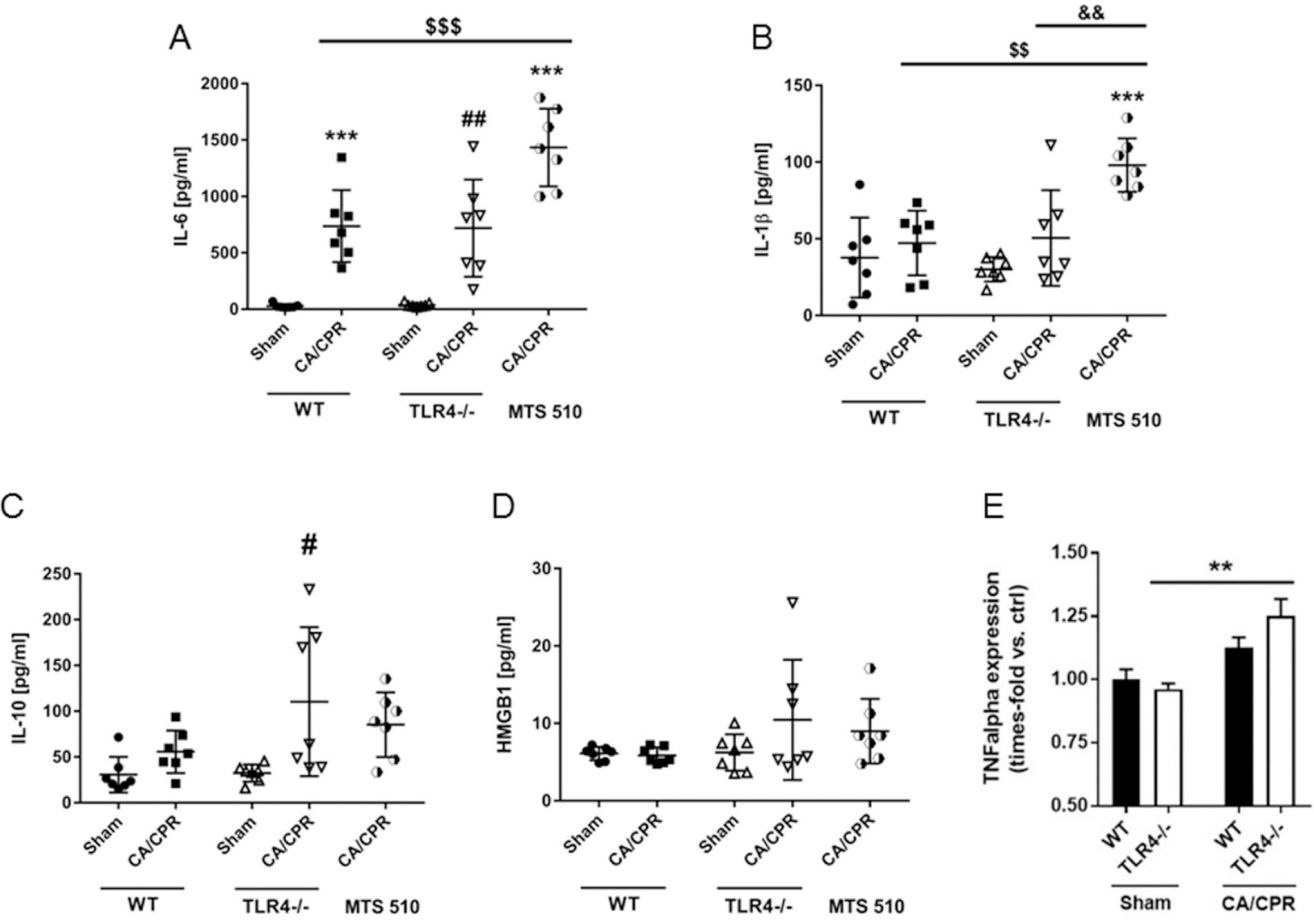
Cytokine plasma and cerebral TNFalpha levels 8h after CA/CPR. In separate sets of experiments, (A) interleukin-6 (IL-6), (B) interleukin-1β (IL-1β), (C) interleukin-10 (IL-10), (D) high mobility group box 1 (HMGB1) plasma levels were assessed in wild type (WT), TLR4^-/-^ and mice treated with TLR4 blocking antibody MTS510.and (E) cerebral TNFalpha expression was analyzed in WT and TLR4^-/-^ mice. **P<0.01, ***P<0.001, #P<0.05, ##P<0.01, $ $P<0.01, $ $ $P<0.001 and &&P<0.01 vs. WT sham control or as indicated. One-way ANOVA/Bonferroni.

In separate sets of experiments, we determined the levels of interleukin-6, interleukin-1β, interleukin-10 and high mobility group box 1 (HMGB1) in sham operated and mice after CA/CPR. Interleukin-6 levels increased significantly and comparably in wild type and TLR4^-/-^ mice after CA/CPR (736.2±318.8 vs. 27.55±18.99pg/mL in wild type and 718.9±431 vs. 37.3pg/mL in TLR4^-/-^ mice, P<0.001 and P<0.01 vs. sham treated animals, respectively, Fig 3A). Resuscitated wild type mice treated with the TLR4 blocking antibody MTS 510 showed twice as high interleukin-6 levels compared to resuscitated wild type mice (1434±344 pg/mL, P<0.001). Interleukin-1β plasma concentrations did not increase in neither resuscitated wild type nor resuscitated TLR4^-/-^ mice compared to sham treated animals of the respective genotype (47.22±21.08 vs. 37.73±26.02pg/mL in wild type and 50.54±31.2 vs. 30.09±7.9pg/mL in TLR4^-/-^ mice, Fig 3B). In contrast, interleukin-1β levels doubled in MTS 510 treated wild type animals after resuscitation (98±17.46pg/mL, P<0.001 vs. wild type sham treated mice and P<0.01 vs. resuscitated wild type and TLR4^-/-^ mice). Interleukin-10 levels increased after CA/CPR only in TLR4^-/-^ mice (110.4±81.38pg/mL vs. 32.37±9.4 pg/mL, P<0.05, Fig 3C). HMGB1 levels were neither increased in wild type animals nor TLR4^-/-^ nor MTS 510 treated wild type mice after resuscitation (Fig 3D). To further characterize the degree of neuroinflammation after CA/CPR, cerebral TNFalpha expression levels were compared in TLR4^-/-^ vs. WT mice after 8h. TNFalpha levels increased significantly only in resuscitated TLR4^-/-^ mice compared to sham-operated TLR4^-/-^ mice (1.25±0.07-fold, P<0.01) but not in WT mice (1.12±0.04-fold, P = 0.117) (Fig 3E).

### Neurofunctional outcome after cardiac arrest and resuscitation

Within the first 5 days after CA/CPR, the level of consciousness, corneal reflex, respiration, righting reflex, coordination and physical activity was scored each day using the Neuro Score[21,22] Wild type mice reached a score of 11 [9–12] on day 1 and a score of 12 out of 12 possible points on day 2 and the following days (Table 2). TLR4^-/-^ mice reached a score of 10 [9–11] on day 1 after CA/CPR and 11 [10–12] on day 2. Beginning on day 3, a full score was reached. MTS 510 treated mice reached a score of 9.5 [8–10.5] on day 1 and 10 [8–12] on day 3 after CA/CPR and achieved full score beginning on day 3 after CA/CPR. There were no statistically significant differences between the Neuro Score achieved between groups.

**Table 2:** Neuro score.

	WT	TLR4^-/-^	WT + MTS 510
	n = 38	n = 37	n = 15
(points)			
Day 1	11 [9 – 12]	10 [9 – 11]	9,5 [8 – 10,5]
Day 2	12 [10 – 12]	11 [10 – 12]	10 [8 – 12]
Day 3	12 [11 – 12]	12 [11 – 12]	12 [5 – 12]
Day 5	12 [12 – 12]	12 [12 – 12]	12 [11 – 12]

The RotaRod Test was used to assess motor function as time of the ability to balance on a rotating cylinder (Table 3)[22,23]. Compared to their baseline ability to balance for a full turn of 15min (900sec) on the rotating rod before CA/CPR, mice of all groups were severely impaired on the first and second day after CA/CPR. TLR4^-/-^ showed full recovery of their ability to balance on the rod on day 4 after CA/CPR, while wild type mice remained impaired until day 4. Analysis of the ability of MTS 510 treated wild type mice to balance on the rod is limited due to less animals included in this treatment group and high mortality rates. However, the numbers reveal a persistent impairment of antibody treated mice until day 5 after CA/CPR. No significant differences between groups were observed for any given day after CA/CPR.

**Table 3:** Rota rod test.

	WT	TLR4^-/-^	WT + MTS 510
	n = 38	n = 37	n = 15
(time [s])			
Day 0	851 [666–900]	900 [885–900]	900 [720–900]
Day 1	40,5 [5 – 603]	46,5 [8 – 324]	18,5 [2 – 90]
Day 2	348 [90–517]	183 [37–309]	180 [24 – 317]
Day 3	401 [138–734]	664 [231–881]	236 [31 – 384]
Day 4	610 [227–900]	900 [723–900]	312 [125–589]
Day 5	810 [578–894]	900 [696–900]	531 [225–781]

Remembrance and spatial learning ability were assessed using the Water Maze test (Table 4). Before CA/CPR, mice were trained to find a rescuing platform in a tank filled with milky water. During the training phase of 5 consecutive days, significant shortening of the time required to find the hidden platform was observed in all groups. As the Water Maze test is physically challenging for the animals, we used a scoring system to identify mice after CA/CPR capable of participating in the test. The requirements for participation in the Water Maze test included achieving full Neuro Score, the completion of one of three attempts to balance on the rod for 5min without falling, recovery of body weight to values prior CA/CPR and the absence of signs of disturbance. There were no significant differences among groups regarding the number of animals capable of participating in the test or the day after CA/CPR these requirements were reached. After CA/CPR, mice of all groups required more time to find the hidden platform again. Within two days of further training, mice of all groups reached the average amount of time needed to rescue themselves onto the platform as needed before CA/CPR. When the position of the platform was then altered, mice of all groups exhibited comparable ability to learn the new position reflected by reduced time required to find the hidden platform.

**Table 4:** Water maze test.

			WT	WT+MTS 510	TLR4^-/-^
			n = 38	n = 15	n = 37
**training phase before CA/CPR—first position of escape platform**					
first attempt prior CA/CPR	day -5	time [s]	18 [9 – 35]		13 [7 – 36]
last attempt prior CA/CPR	day -1	time [s]	4 [2 – 18]		6 [3 – 17]
**after CA/CPR—first position of escape platform (remembrance of position)**					
first attempt after CA/CPR	day X	time [s]	8 [3 – 18]	7 [4 – 26]	15 [7 – 28]
last attempt after CA/CPR	day X+2	time [s]	6 [3 – 14]	3 [2 – 4]	8 [5 – 16]
**after CA/CPR—alternative position of escape platform (new spatial learning)**					
first attempt new position	day 10	time [s]	12 [6 – 28]	9 [6 – 23]	10 [3 – 22]
end of training phase	day 15	time [s]	8 [1 – 12]	3 [3 – 4]	9 [3 – 22]
**participation in the Water Maze Test after CA/CPR**					
paticipants after CA/CPR		n [%]	15 [39,5]	5 [33,3]	19 [51,4]
earliest day of participation after CA/CPR (day X)		day	5 [4 – 6]	4 [3 – 5]	6 [4 – 7]
**Requirements for participation in the Water Maze Test after CA/CPR**					
to get full marks for the NeuroScore				12 of 12 points	
to complete one of three attempts in RotaRod-Test without falling from the rod				[300 sec]	
show recovery of bodyweight					
no signs of disturbance					

## Discussion

Toll like receptors (TLRs) have been implicated in ischemic brain injury. They are expressed on neurons, microglia, astrocytes and oligodendrocytes and their activation by endogenous or exogenous ligands during ischemia and reperfusion results in a rapid induction of inflammatory signaling cascades initiating the synthesis and release of pro-inflammatory mediators [13]. Inflammation is one of the hallmarks of hypoxic ischemic encephalopathy [27] and experimental studies suggest that the absence of either TLR2 or TLR4 is associated with protection against ischemic brain injury [28]. We have previously investigated the impact of genetic deficiency or pharmacological blockade of TLR2 on survival and neurofunctional outcome after CA/CPR [16]. TLR2^-/-^ and TLR2 antibody treated mice exhibited increased survival, achieved a higher NeuroScore, exhibited no or only mild impairment when balancing on the rotating rod after CA/CPR and TLR2 antibody treated mice better preserved their ability to learn and remember after CA/CPR than wild type mice. In the present study, mice genetically deficient of TLR4 showed a tendency towards increased survival after CA/CPR but the difference in survival compared to wild type mice did not reach a level of statistical significance. Based on the given survival data, prospective biostatistical analyzes performed by a statistician revealed that more than 100 mice per group would be required to subject to CA/CPR (WT vs. TLR4^-/-^) to achieve statistically significant differences which would have been beyond the scope of this pre-clinical observational study. Other groups have identified protective effects of TLR4 deficiency or administration of TLR4 inhibiting agents in mouse models of CA/CPR analyzing groups of comparable sizes [11,29]. For example, Xu and colleagues conducted potassium-induced cardiac arrest in TLR4 mutant mice (C3H/HeJ) for 3 min followed by CPR. Mice that did not exhibit ROSC were excluded from the study. The authors assessed general activity and anxiety-like behavior in an open field apparatus on day 3 after CA/CPR. Within the first 72h after CA/CPR, TLR4 mutant mice exhibited increased survival compared to controls (C3H/HeN). Plotting of survival data from our present study shows that indeed TLR4^-/-^ mice may exhibit a tendency towards increased survival rates compared to wild type controls within the first 3 days after CA/CPR but the majority of animals was lost after day 3 and lethal complications continued to occur until day 24 after CA/CPR. Analogous, Andresen et al. postulated beneficial short- but not long-term effects of MTS510 blocking TLR4 in a model of experimental stroke [20]. In the present and our previous studies, the length of an observation period of 28 days was chosen to evaluate neurofunction in a time frame of utmost clinical relevance to patients after CA/CPR. For patients, impairment of higher cognitive functions such as intellect and memory represent the main obstacles for a return to an independent way of life after discharged from hospital [4]. Previously, similar performance of the same set of neurofunctional tests assessing balance, coordination and locomotion in addition to spatial learning and remembrance beyond day 3 after CA/CPR further identified improved neurological outcome in mice without or reduced TLR2 signaling ability [16]. In the present study, no advantage in neurological outcome was identified for either mice genetically deficient of TLR4 or wild type mice treated with TLR4 blocking antibodies compared to wild type controls further supporting the conclusion that TLR4 may not serve as a suitable target to ameliorate ischemia and reperfusion induced cerebral injury. In a study assessing outcome after traumatic brain injury, authors had observed TLR4 upregulation in hippocampal astrocytes and neurons and the local application of shRNA silencing TLR4 was associated with alleviation of hippocampal neuronal damage, reduced brain edema formation and reduced neurological deficits in rats assessed by histology [30]. Although we did not characterize CA/CPR induced changes to cerebral tissue morphology in the present study (this was primarily avoided for the sake of a 28-day availability of mice for neurological assessment), we have previously identified CA/CPR induced neuronal cell apoptosis in the CA1/CA2 region of the hippocampus in our model by histology and NMR-based assessments highly correlated with neurofunctional outcome [16]. Thus and although only speculative, we hypothesize TLR4 deficiency or treatment with TLR4 blocking antibodies did not affect the degree of cerebral tissue damage induced by CA/CPR in the present study.

Although TLR4 and other TLR2 contribute to the ongoing inflammatory reaction during ischemia and reperfusion, “priming” of TLR activated signaling pathways is also suggested to confer neuroprotection [31]. For example, brief occlusion of the middle cerebral artery prior to extended occlusion resulted in reduced ischemia induced brain injury and these effects were, at least in part, dependent on TLR4 [32]. Protective effects against cerebral ischemia were also observed by administration of TLR4 ligands. TLRs are expressed on brain parenchymal cells such as neurons and glia but also on a variety of hematopoietic cells and the endothelium. Protective effects of exogenously administered TLR ligands against ischemic brain injury were observed independently of the ligands ability to cross the blood brain barrier, suggesting the TLR mediated effects on brain parenchymal cells may depend on indirect mechanisms. In chimeric mouse models, neither depletion of TLR9 on hematopoietic nor vascular or brain parenchymal cells alone conferred comparable neuroprotection as the ubiquitous TLR deficiency [33]. The results of the present study may thus display a combination of both limiting potentially deleterious pro-inflammatory effects while on the other hand reducing protective effects mediated by TLR4. For preconditioning strategies, it is essential that the sub-deleterious stimulus is provided before the actual injury occurs. In this regard, application of a small molecule inhibitor of TLR4 administered *before* CA/CPR conducted in a rat model was associated with reduced neuronal degeneration [11]. However this order of events, i.e. the administration of a pharmacological agent prior to CA, is not representative for the clinically relevant scenario of out-of-hospital CA and therefore of limited translational implication.

Interestingly, TLR4 deficient mice exhibited a cytokine profile after CA/CPR rather opposite than the expected, since this genotype has been associated with reduced expression of proinflammatory genes [34]. TLR4 deficient mice showed higher interleukin-6 levels compared to wild type controls and higher interleukin-10 levels although TLR4 signaling is known to be an important regulator of interleukin-6 and interleukin-10 production at the transcriptional as well as post-transcriptional level. However studies observed that the inflammatory reaction in a given model was not substantially ameliorated by TLR4 deficiency: Instead, increased levels of either cytokine where encountered [35] or TLR4 deficiency promoted the activation of alternative inflammatory pathways which is supported by our results showing increased levels of TNFalpha expression in cerebral tissues 8h after CA/CPR in TLR4^-/-^ but not WT mice. This suggests that TLR4 deficiency results in a phenotype that is not particularly characterized by an overall limitation of the inflammatory activity [36]. With respect to our previous study investigating the effects of TLR2 deficiency or TLR2 functional inhibition in this model of CA/CPR, we may conclude that assessment of the inflammatory situation 8h after CA/CPR by measurement of interleukin-6, -1β or -10 or cerebral TNFalpha expression does overall not suffice for predicting survival or neurofunctional outcome, since wild type mice treated with antibodies against TLR2 or TLR4 exhibited comparable increase in interleukins but showed opposite outcome compared to control treated wild type mice.

Strategies for the identification of targets for therapy in preclinical models often failed when translated into a clinical setting relevant to patient outcome. Although numerous obstacles persist, assessments and preclinical model design should aim to resemble the closest as possible situation to clinical scenarios. With the present study, we aimed to conduct CA for a duration relevant to real life scenarios of out-of-hospital cardiac arrest, where CPR is often not conducted by lay bystanders and the average time for an ambulance to arrive is at best ~10min. We assessed survival during a period of 28 days and found lethal complications until day 24 after CA/CPR, suggesting that shorter observation periods may over- or underestimate effect size. For neurological outcome, we assessed parameters of neuromotor function and higher cognitive abilities such as spatial learning and remembrance in order to address the leading neurological limitations of survivors after CA/CPR and discharge from hospital. Although we did not assess additional parameters such as cardiac function and limited a pharmacological approach of TLR4 inhibition to the use of antibodies only, we conclude that we could not identify TLR4 as a suitable target for alleviating post resuscitation mortality or neurofunctional impairment after CA/CPR.

## Supporting information

S1 Fig Verification of the *Tlr4^Lps-del^* spontaneous mutation corresponding to a shortened mRNA product of 140 instead of 390bp. (TIF)

S2 Fig Reduced interleukin-6 (IL-6) and interleukin-10 (IL-10) levels in TLR4 knock-out mice 8h after lipopolysaccharide injection. *P<0.05, Two-way ANOVA/Bonferroni.
(TIF)

S3 Fig Forest plot for Cox Proportional Hazards Model. WT mice are used as the reference with the hazard ratio of 1. Hazard ratios for TLR4 and MTS 510 groups are plotted together with their 95% confidence intervals and group specific p-values. No significant differences between the groups were detected.
(TIF)

## References

[1] Benjamin EJ, Virani SS, Callaway CW, Chamberlain AM, Chang AR, Cheng S, et al (2018) Heart disease and stroke statistics—2018 update: a report from the American Heart Association. Circulation 137: e67–e492. 10.1161/CIR.0000000000000558 29386200

[2] Gräsner J-T, Lefering R, Koster RW, Masterson S, Böttiger BW, Herlitz J, et al (2016) EuReCa ONE 27 Nations, ONE Europe, ONE Registry: A prospective one month analysis of out-of-hospital cardiac arrest outcomes in 27 countries in Europe. Resuscitation 105: 188–195. 10.1016/j.resuscitation.2016.06.004 27321577

[3] Fertl E, Vass K, Sterz F, Gabriel H, Auff E (2000) Neurological rehabilitation of severely disabled cardiac arrest survivors. Part I. Course of post-acute inpatient treatment. Resuscitation 47: 231–239. 11114452 10.1016/s0300-9572(00)00239-2

[4] Pußwald G, Fertl E, Faltl M, Auff E (2000) Neurological rehabilitation of severely disabled cardiac arrest survivors. Part II. Life situation of patients and families after treatment. Resuscitation 47: 241–248. 11114453 10.1016/s0300-9572(00)00240-9

[5] Püttgen HA, Pantle H, Geocadin RG (2009) Management of cardiac arrest patients to maximize neurologic outcome. Current opinion in critical care 15: 118–124. 10.1097/MCC.0b013e328326077c 19578322

[6] Laver S, Farrow C, Turner D, Nolan J (2004) Mode of death after admission to an intensive care unit following cardiac arrest. Intensive care medicine 30: 2126–2128. 10.1007/s00134-004-2425-z 15365608

[7] Niemann JT (1984) Differences in cerebral and myocardial perfusion during closed-chest resuscitation. Annals of emergency medicine 13: 849–853. 10.1016/s0196-0644(84)80458-8 6476554

[8] Maramattom BV, Wijdicks EF (2005) Postresuscitation encephalopathy: current views, management, and prognostication. The neurologist 11: 234–243. 10.1097/01.nrl.0000159985.07242.22 15989696

[9] Miyamoto O, Auer R (2000) Hypoxia, hyperoxia, ischemia, and brain necrosis. Neurology 54: 362–362. 10.1212/wnl.54.2.362 10668697

[10] Oda Y, Tsuruta R, Fujita M, Kaneda K, Kawamura Y, Izumi T, et al (2012) Prediction of the neurological outcome with intrathecal high mobility group box 1 and S100B in cardiac arrest victims: a pilot study. Resuscitation 83: 1006–1012. 10.1016/j.resuscitation.2012.01.030 22306257

[11] Shi X, Li M, Huang K, Zhou S, Hu Y, Pan S, et al (2017) HMGB1 binding heptamer peptide improves survival and ameliorates brain injury in rats after cardiac arrest and cardiopulmonary resuscitation. Neuroscience 360: 128–138. 10.1016/j.neuroscience.2017.07.052 28778700

[12] Krupinski J, Ethirajan P, Font MA, Turu MM, Gaffney J, Kumar P, et al (2007) Changes in hyaluronan metabolism and RHAMM receptor expression accompany formation of complicated carotid lesions and may be pro-angiogenic mediators of intimal neovessel growth. Biomarker insights 2: 361–367. PMC2717833 19662229

[13] Wang Y, Ge P, Zhu Y (2013) TLR2 and TLR4 in the brain injury caused by cerebral ischemia and reperfusion. Mediators of inflammation 2013:124614 10.1155/2013/124614 23864765 PMC3706022

[14] Asmussen A, Fink K, Busch H-J, Helbing T, Bourgeois N, Bode C, et al (2016) Inflammasome and toll-like receptor signaling in human monocytes after successful cardiopulmonary resuscitation. Critical Care 20: 170 10.1186/s13054-016-1340-3 27260481 PMC4893227

[15] Peberdy MA, Callaway CW, Neumar RW, Geocadin RG, Zimmerman JL, Donnino M, et al (2010) Part 9: post–cardiac arrest care: 2010 American Heart Association guidelines for cardiopulmonary resuscitation and emergency cardiovascular care. Circulation 122: S768–S786. 10.1161/CIRCULATIONAHA.110.971002 20956225

[16] Bergt S, Guter A, Grub A, Wagner NM, Beltschany C, Langner S, et al (2013) Impact of Toll-like receptor 2 deficiency on survival and neurological function after cardiac arrest: a murine model of cardiopulmonary resuscitation. PLoS One 8: e74944 10.1371/journal.pone.0074944 24066159 PMC3774715

[17] Bergt S, Grub A, Wagner S, Engelke H, Nöldge-Schomburg G, Vollmar B, et al (2017) Pravastatin But Not Simvastatin Improves Survival and Neurofunctional Outcome After Cardiac Arrest and Cardiopulmonary Resuscitation. JACC: Basic to Translational Science: 105. 10.1016/j.jacbts.2017.01.009 PMC6113548 30167563

[18] Poltorak A, He X, Smirnova I, Liu M-Y, Van Huffel C, Du X, et al (1998) Defective LPS signaling in C3H/HeJ and C57BL/10ScCr mice: mutations in Tlr4 gene. Science 282: 2085–2088. 10.1126/science.282.5396.2085 9851930

[19] Yang H-Z, Wang J-P, Mi S, Liu H-Z, Cui B, Yan H-M, et al (2012) TLR4 activity is required in the resolution of pulmonary inflammation and fibrosis after acute and chronic lung injury. The American journal of pathology 180: 275–292. 10.1016/j.ajpath.2011.09.019 22062220

[20] Andresen L, Theodorou K, Grünewald S, Czech-Zechmeister B, Könnecke B, Lühder F, et al (2016) Evaluation of the therapeutic potential of anti-TLR4-antibody MTS510 in experimental stroke and significance of different routes of application. PloS one 11: e0148428 10.1371/journal.pone.0148428 26849209 PMC4746129

[21] Abella BS, Zhao D, Alvarado J, Hamann K, Hoek TLV, Becker LB. (2004) Intra-arrest cooling improves outcomes in a murine cardiac arrest model. Circulation 109: 2786–2791. 10.1161/01.CIR.0000131940.19833.85 15159295

[22] Neigh GN, Glasper ER, Kofler J, Traystman RJ, Mervis RF, Bachstetter A, et al (2004) Cardiac arrest with cardiopulmonary resuscitation reduces dendritic spine density in CA1 pyramidal cells and selectively alters acquisition of spatial memory. European Journal of Neuroscience 20: 1865–1872. 10.1111/j.1460-9568.2004.03649.x 15380008

[23] Hutchens MP, Nakano T, Dunlap J, Traystman RJ, Hurn PD, Alkayed NJ (2008) Soluble epoxide hydrolase gene deletion reduces survival after cardiac arrest and cardiopulmonary resuscitation. Resuscitation 76: 89–94. 10.1016/j.resuscitation.2007.06.031 17728042 PMC2585367

[24] Neigh GN, Kofler J, Meyers JL, Bergdall V, La Perle KM, Traystman RJ, et al (2004) Cardiac arrest/cardiopulmonary resuscitation increases anxiety-like behavior and decreases social interaction. Journal of Cerebral Blood Flow & Metabolism 24: 372–382. 10.1097/01.WCB.0000112323.75217.B4 PMC1363744 15087706

[25] Decker MW, McGaugh JL (1989) Effects of concurrent manipulations of cholinergic and noradrenergic function on learning and retention in mice. Brain research 477: 29–37. 10.1016/0006-8993(89)91391-7 2702490

[26] Nicholson A, Malcolm RD, Russ PL, Cough K, Touma C, Palme R, et al (2009) The response of C57BL/6J and BALB/cJ mice to increased housing density. Journal of the American Association for Laboratory Animal Science 48: 740–753. 19930822 PMC2786928

[27] Liu F, Mccullough LD (2013) Inflammatory responses in hypoxic ischemic encephalopathy. Acta Pharmacologica Sinica 34: 1121 10.1038/aps.2013.89 23892271 PMC3764334

[28] Tang S-C, Arumugam TV, Xu X, Cheng A, Mughal MR, Jo DG, et al (2007) Pivotal role for neuronal Toll-like receptors in ischemic brain injury and functional deficits. Proceedings of the National Academy of Sciences 104: 13798–13803. 10.1073/pnas.0702553104 PMC1959462 17693552

[29] Xu L, Zhang Q, Zhang Q-S, Li Q, Han J-Y, Sun P (2015) Improved survival and neurological outcomes after cardiopulmonary resuscitation in toll-like receptor 4-mutant mice. Chinese medical journal 128: 2646 10.4103/0366-6999.166024 26415804 PMC4736870

[30] Jiang H, Wang Y, Liang X, Xing X, Xu X, Zhou C (2017) Toll-like receptor 4 knockdown attenuates brain damage and neuroinflammation after traumatic brain injury via inhibiting neuronal autophagy and astrocyte activation. Cellular and molecular neurobiology: 1–11. 10.1007/s10571-016-0330-y 29222622 PMC11481957

[31] Gesuete R, Kohama SG, Stenzel-Poore MP (2014) Toll-like receptors and ischemic brain injury. Journal of Neuropathology & Experimental Neurology 73: 378–386. 24709682 10.1097/NEN.0000000000000068 PMC4115675

[32] Pradillo JM, Fernández‐López D, García‐Yébenes I, Sobrado M, Hurtado O, Moro MA, et al (2009) Toll‐like receptor 4 is involved in neuroprotection afforded by ischemic preconditioning. Journal of neurochemistry 109: 287–294. 10.1111/j.1471-4159.2009.05972.x 19200341

[33] Packard AE, Leung PY, Vartanian KB, Stevens SL, Bahjat FR, Stenzel-Poore MP (2012) TLR9 bone marrow chimeric mice define a role for cerebral TNF in neuroprotection induced by CpG preconditioning. Journal of Cerebral Blood Flow & Metabolism 32: 2193–2200. 23010947 10.1038/jcbfm.2012.140 PMC3519417

[34] Dvoriantchikova G, Barakat DJ, Hernandez E, Shestopalov VI, Ivanov D (2010) Toll-like receptor 4 contributes to retinal ischemia/reperfusion injury. Molecular vision 16: 1907 21031135 PMC2956702

[35] Thaete LG, Qu X-W, Jilling T, Crawford SE, Fitchev P, Hirsch E, et al (2013) Impact of toll-like receptor 4 deficiency on the response to uterine ischemia/reperfusion in mice. Reproduction: REP-12-0433. 10.1530/REP-12-0433 23509372

[36] Orr JS, Puglisi MJ, Ellacott KL, Lumeng CN, Wasserman DH, Hasty AH (2012) Toll-like receptor 4 deficiency promotes the alternative activation of adipose tissue macrophages. Diabetes: DB_111595. 10.2337/db11-1595 PMC3478520 22751700

