# Supplementary figures and images for: Toll-like receptor 4 deficiency or inhibition does not modulate survival and neurofunctional outcome in a murine model of cardiac arrest and resuscitation
Source: PLoS One. 2019 Aug 1;14(8):e0220404. doi: 10.1371/journal.pone.0220404 (PMC6675321; doi:10.1371/journal.pone.0220404)

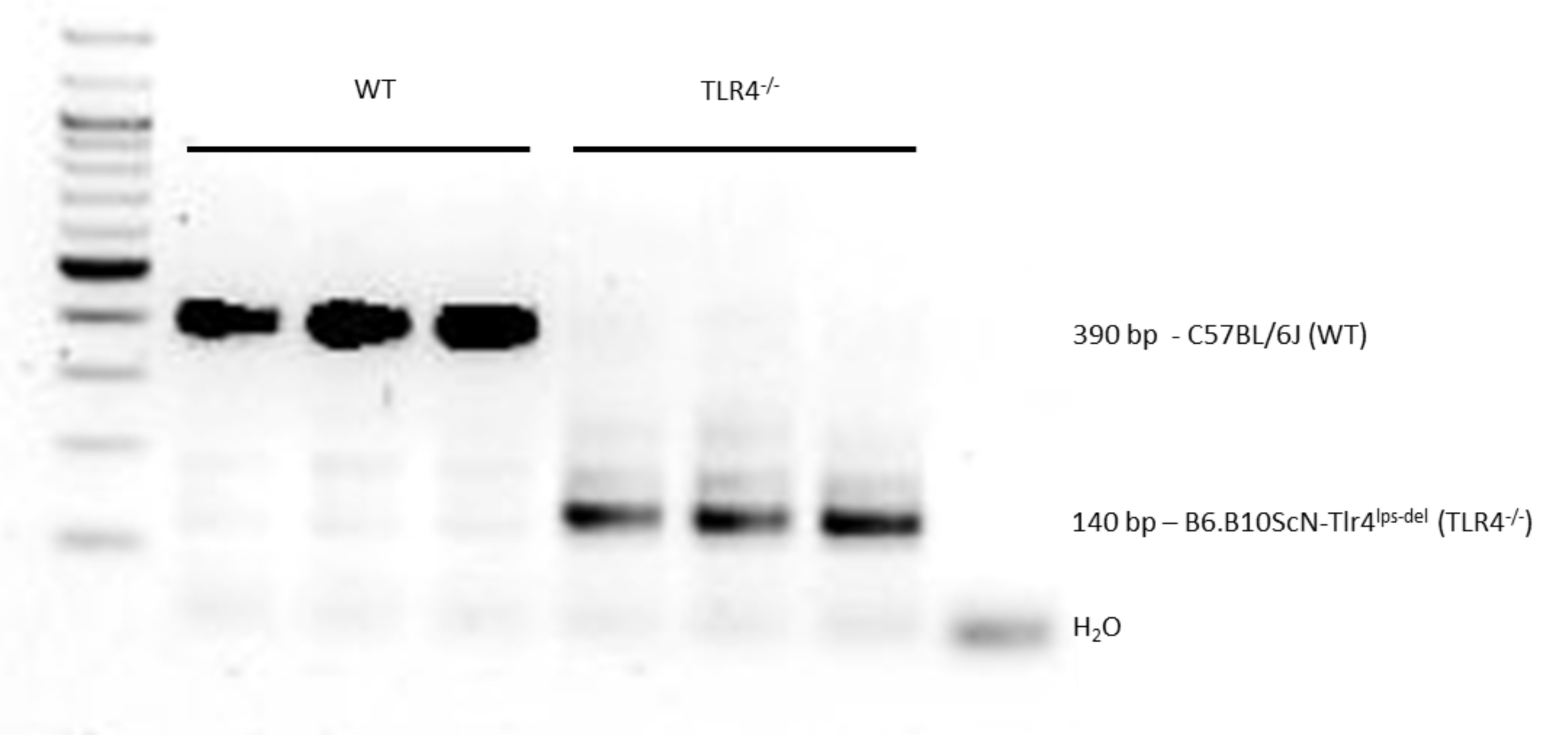

Supplement: S1 Fig — (TIF) [file pone.0220404.s001.tif]

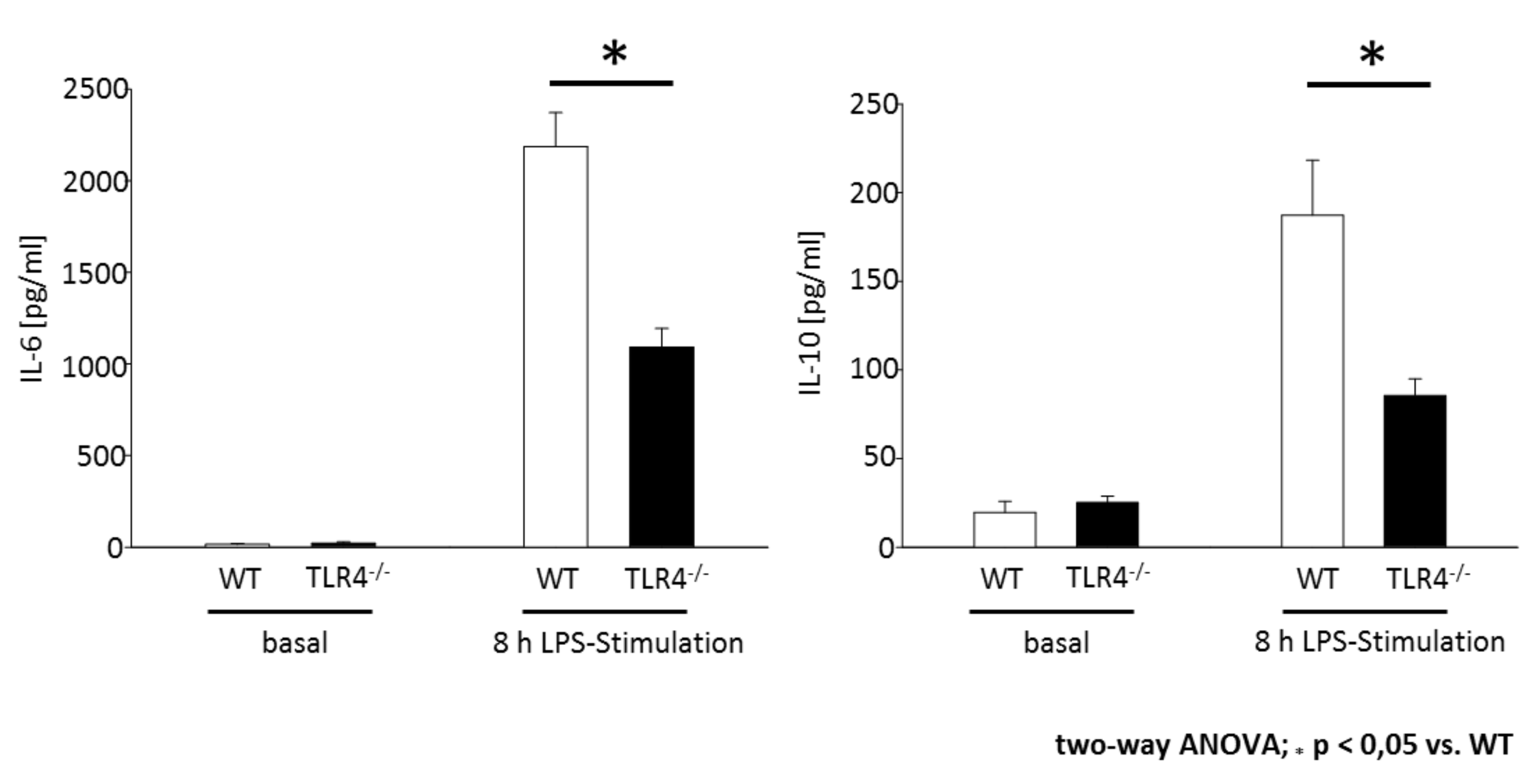

Supplement: S2 Fig — *P<0.05, Two-way ANOVA/Bonferroni. (TIF) [file pone.0220404.s002.tif]

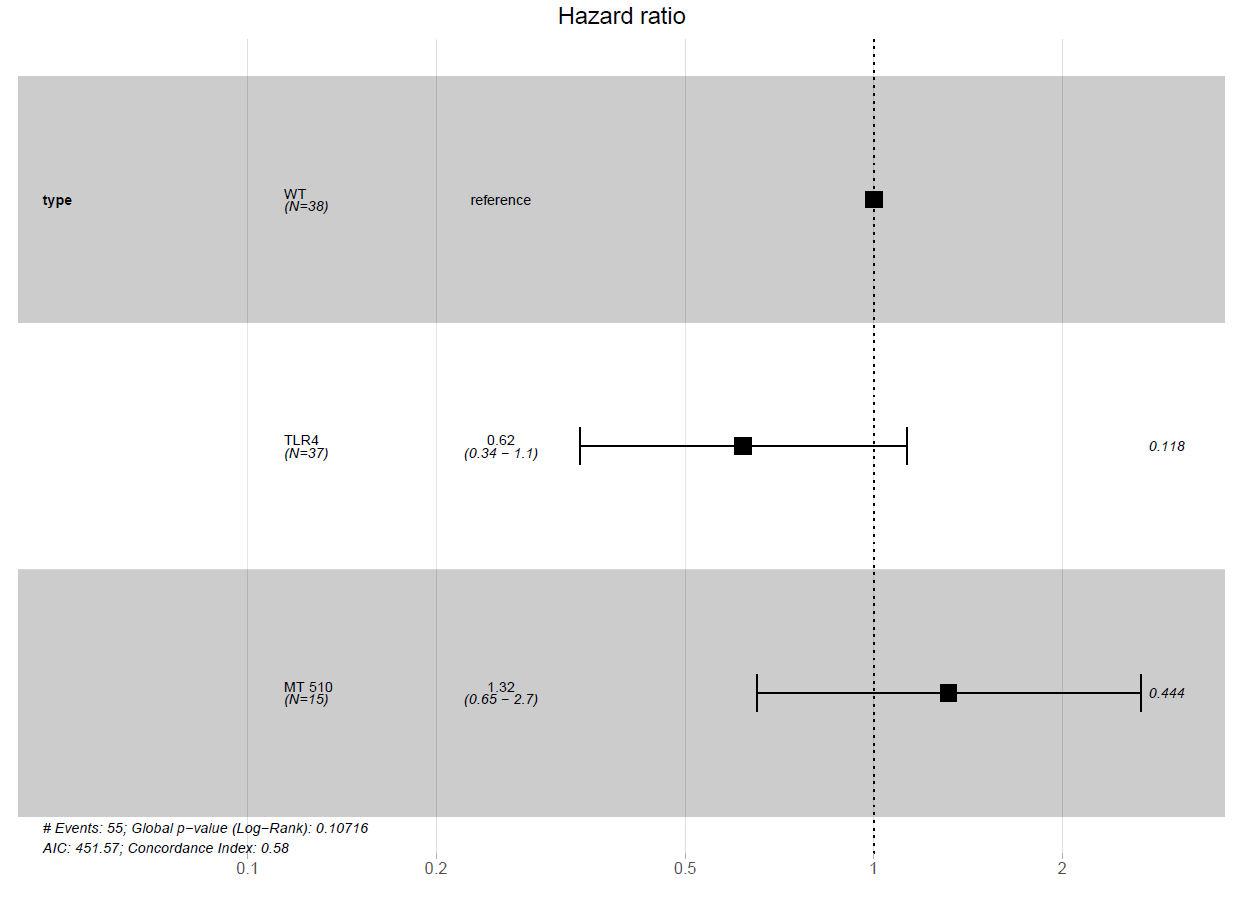

Supplement: S3 Fig — WT mice are used as the reference with the hazard ratio of 1. Hazard ratios for TLR4 and MTS 510 groups are plotted together with their 95% confidence intervals and group specific p-values. No significant differences between the groups were detected. (TIF) [file pone.0220404.s003.tif]
